# Enhancing Youth Mental Health Through Virtual Lifestyle Behavior Change Support: A Pilot Feasibility Trial

**DOI:** 10.3390/children13020163

**Published:** 2026-01-23

**Authors:** Meaghan Halle Smith, Patricia E. Longmuir, Marjorie Robb, Mark L. Norris, Miranda DiGasparro, Kaitlin Laurie, Natasha Baechler, Natasha McBrearty, Kimberly Courtney, Fiona Cooligan, Paula Cloutier, Clare Gray

**Affiliations:** 1 Faculty of Medicine, University of Ottawa, Ottawa, ON K1N 6N5, Canada; 2 Children’s Hospital of Eastern Ontario Research Institute, Ottawa, ON K1H 8L1, Canada; 3 Crossroads Children’s Mental Health Centre, Ottawa, ON K2C 3J2, Canada; 4 Children’s Hospital of Eastern Ontario, Ottawa, ON K1H 8L1, Canada

**Keywords:** pediatric mental health, adolescents, digital intervention, health promotion, lifestyle change, anxiety, depression

## Abstract

**Highlights:**

**What are the main findings?**

- Approximately 25% of youth with mental distress engaged with HELP e-resources.
- Engaged youth reported favorable mental health and lifestyle patterns over 3 months.
- Screen time habits appeared most responsive to behavior change content.

**What are the implications of the main findings?**

- HELP may represent a feasible, community-based support to bridge gaps in pediatric mental health care.
- Youth with milder baseline mental health appeared more likely to benefit.

**Abstract:**

**Background**: Among many deleterious effects on the well-being of children and youth, the COVID-19 pandemic contributed to a surge in youth mental health distress. This, coupled with pre-existing prolonged wait times for mental health care, highlighted the need for accessible community-based mental health supports. The Healthy Living Project (HELP) is a virtual lifestyle change support program aimed at promoting positive lifestyle changes and improved mental well-being among youth with mental distress. A pilot feasibility study explored youth engagement with HELP e-resources, and preliminary mental health and lifestyle measures over a 3-month period. **Methods**: Youth were enrolled in a 3-month pilot of the HELP e-resource. Feasibility metrics (recruitment, retention, and platform engagement) were documented, while exploratory self-reported data on emotional and behavioral difficulties, youth quality of life, sedentary behavior (screen time), sleep hygiene, and physical activity were assessed at baseline and 3 months. **Results**: Twenty-three youth (mean age 15.7 years, SD 1.7) completed baseline assessments and started the intervention, with ten participants retained by the end of the study. Compared with non-completers (*n* = 13), study completers (*n* = 10) tended to report higher quality of life and healthier habits (lower screen time, improved sleep hygiene, and higher activity). Ongoing access to HELP over 3 months was associated with suggestive trends toward improvement in emotional and behavioral difficulties and sleep hygiene. Engaged participants who received screen time education tended to report lower screen times as compared to unengaged counterparts. **Conclusions**: This study provides early insights into the implementation and acceptability of HELP e-resources among youth experiencing mental distress, with suggestive trends toward potential benefit. Low recruitment and high attrition preclude definitive conclusions, and the findings should be interpreted as exploratory. Lessons from this pilot will inform the design of a subsequent trial to more rigorously evaluate feasibility and the potential impact of HELP on youth with mental distress.

## 1. Introduction

The COVID-19 pandemic amplified youth mental distress [1] and mental health-related hospitalizations [2]. Even prior to the pandemic, youth in Ontario faced wait times of up to 2.5 years for mental health care [3]. Combined with ongoing specialized mental health support shortages, these delays highlight a clear and urgent need for enhanced community-based interventions to help ameliorate distress in children and youth.

Lifestyle behaviors, including increased screen time [4], decreased physical activity [5] and disrupted sleep [6] have all been shown to have a deleterious impact on youth mental well-being. Although lifestyle changes have shown beneficial effects on mental health outcomes in adult populations [7,8], research supporting their effectiveness among children and youth is limited.

Over the past decade, digital mental health interventions have emerged as a potential strategy to improve mental health outcomes in youth, with examples including Bite Back [9] and Appa Health App [10]. However, existing interventions have not focused on addressing mental distress through modifiable lifestyle behaviors. The Healthy Lifestyle Project (HELP) is an interactive, virtual lifestyle change support tool designed to help youth improve health-related behaviors such as screen time habits, physical activity, and good sleep hygiene. HELP assesses readiness for change and provides tailored, goal-driven programs, with more than 70 options available to meet youth’s individual needs. In addition to offering step-by-step guidance for improving lifestyle behaviors, HELP provides educational content to support youth’s understanding of the connection between their health behaviors and mental health. The platform is intended for use in home and community settings and may be particularly relevant to youth awaiting access to more formal mental health services.

This pilot study aimed to assess the feasibility of youth engagement with the HELP platform and to obtain preliminary data on patterns of resource engagement and associated lifestyle and mental health outcomes. It was not designed to evaluate the efficacy of the intervention. It was hypothesized that engagement with the platform would be feasible and active e-resource use would lead to favorable trends in lifestyle behaviors and mental health-related outcomes.

## 2. Methods

### 2.1. Participants

**Figure 1:**
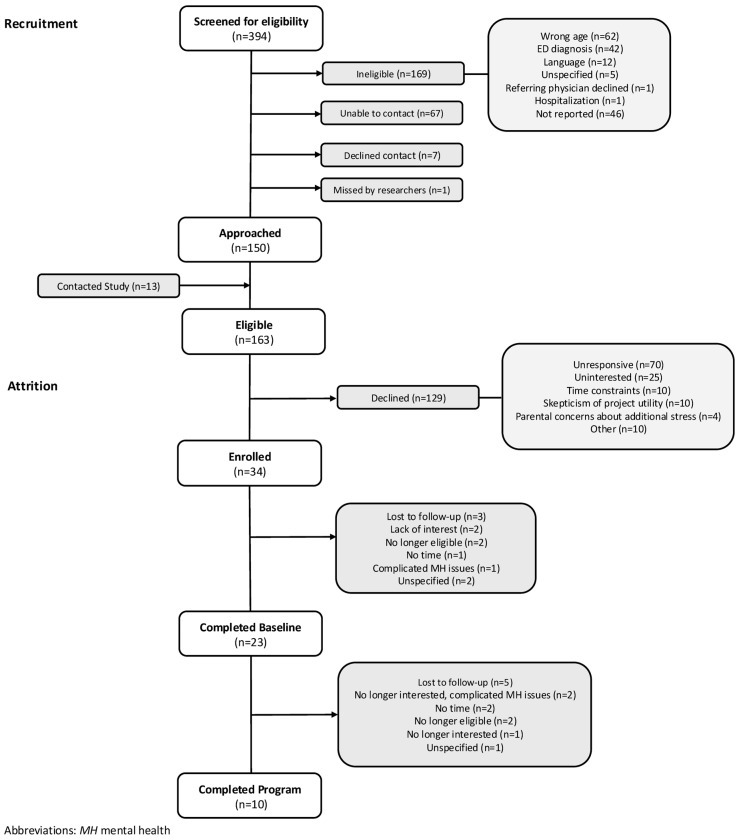
CONSORT diagram for HELP recruitment, enrollment, and attrition.

Youth aged 13–20 years old who sought mental health services between October 2021 and April 2023 were eligible to participate in the study. Recruitment occurred via outpatient mental health waitlists, youth mental health groups, and through the regional centralized youth mental health service intake. Hospitalized youth and those referred for or diagnosed with concerns related to disordered eating or eating disorders were excluded. Reasons for withdrawal and loss to follow-up are detailed in the CONSORT diagram for HELP recruitment, enrollment, and attrition (Figure 1).

This study received approval from the Children’s Hospital of Eastern Ontario Research Ethics Board (REB file #: 21/85X). Verbal informed consent was obtained from participants.

### 2.2. The HELP E-Resource

The HELP e-resources were developed using an integrative, iterative methodology that combined feedback from youth experiencing mental distress (youth hospitalized with mental illness: *n* = 12; youth receiving out-patient support: *n* = 33) and their parents or caregivers (*n* = 18), alongside clinician expertise (*n* = 11) and peer-reviewed evidence. Youth and parents/caregivers provided input on content relevance, usability, and acceptability, ensuring that the platform reflected youth-identified priorities, while also remaining developmentally appropriate and based on current best evidence. Their feedback was continuously incorporated throughout module development, with the content being refined through multiple cycles until satisfaction ratings reached ≥8/10 on a pre-defined 10-point feedback scale (Laurie et al., unpublished results). Once satisfaction ratings achieved 8/10 or higher, the module development was considered complete, and no further changes were made. All modules were complete before the pilot study participants were enrolled.

HELP consists of four core sections: (1) Know Your Habits, (2) Physical Activity, (3) Screen Time, and (4) Sleep. The Know Your Habits section provides tailored feedback based on youths’ baseline assessments, including readiness to change and self-reported lifestyle behavior assessments. Based on these baseline assessments, participants were provided with tailored recommendations to guide engagement throughout the 3-month access period. The Physical Activity, Screen Time, and Sleep sections each include three components: (1) Learn, (2) Pros and Cons, and (3) Goals. Participants who did not recognize a need for change were directed to Learn modules, which explain the relationship between lifestyle behaviors and mental wellness. Youth who expressed ambivalence toward change were guided through Pros and Cons activities designed to explore perceived barriers and facilitators to behavior change. Participants who indicated readiness to change were guided to develop SMART (specific, measurable, achievable, realistic, and timely) behavior change goals. Youth could select from over 70 predefined change plans, each comprising 6–8 structured steps toward a desired goal. Resources to support navigation of setbacks or behavior relapse were provided, along with optional access to a registered kinesiologist for additional support. While participants received tailored recommendations based on baseline assessments, engagement with specific modules was self-directed, and access to module content was not restricted or controlled by the research team. Participants were provided access to the HELP platform for a 3-month period.

The time required to engage with the HELP modules varied by module type and participant-selected activities. Educational (Learn) modules addressing the relationship between lifestyle behaviors and mental health were brief, typically requiring approximately 5 min to complete. Modules focused on exploring ambivalence toward behavior change through Pros and Cons activities required more time, often taking 20 min or longer depending on the depth of reflection and discussion. Behavior change goals consisted of structured change plans comprising 6–8 sequential steps. These steps were intentionally not time-limited, enabling youth to progress at their own pace based on individual capacity and circumstances. The time required to complete individual steps varied substantially, ranging from brief activities (i.e., logging bedtime across multiple days) to more time-intensive behaviors (i.e., engaging in 30 min of physical activity on multiple days within a week).

### 2.3. Study Measures and Data Collection

Demographic data, including age and gender, were collected at baseline through a self-report questionnaire. Information regarding participants’ diagnoses was extracted from medical charts, if available. The total Strengths and Difficulties score (SDQ; Reliability: Cronbach’s α = 0.73) [11], a validated screening measure for emotional and behavioral difficulties in children and youth with mental health challenges [12], served as the primary outcome of interest. Pre- and post-assessments were completed to explore preliminary signals of change in well-being and lifestyle behaviors following access to the HELP virtual lifestyle change support. Baseline and post-intervention lifestyle behaviors were evaluated using validated questionnaires completed by youth, with or without a parent/guardian depending on participant capacity. Questionnaires were administered and scored using the Research Electronic Data Capture platform [13]. The Youth Quality of Life Instrument total score (YQoL) [14] assessed quality of life (Reliability: Cronbach’s α = 0.77–0.96, ICC = 0.74–0.85 [15]). Sleep hygiene was assessed using the Adolescent Sleep Hygiene Scale (ASHS; Reliability: Cronbach’s α = 0.84) [16]. Average daily screen time was determined by the Adolescent Sedentary Activity Questionnaire (ASAQ; Reliability: ICC = 0.57 to 0.70) [17]. Average daily active time was measured with the Habitual Activity Estimation Scale (Reliability: weekday *g* = 0.75–0.92; weekend *g* = 0.35–0.74) [18].

User activity was tracked automatically through login records and page visits. Engagement analyses focused on the number of visits to the “Physical Activity”, “Screen Time”, and “Sleep” sections of the e-resource. To categorize participants as low- or high-engagement for each lifestyle behavior, youth with ≥10 web page visits to a behavior section during the 3-month access period were considered high-engagement (low-engagement < 10 visits). The ≥10 webpage visit threshold was selected as an exploratory indicator of engagement for descriptive purposes. This cutoff was not theory-driven and does not capture visit duration or content quality, which should be addressed in future studies. For emotional and behavioral difficulties (SDQ) and quality of life (YQoL) indicators, participants were categorized as “engaged” with HELP if they demonstrated high engagement with at least one lifestyle behavior. Participants who did not exhibit high engagement with any behavior were considered “unengaged”. This categorization was utilized because of the flexibility that participants had to focus on one, two, or all three target behaviors. This categorization was used to reflect the flexibility afforded to participants to focus on one, two, or all three target behaviors, and was intended for exploratory and descriptive purposes rather than theory-driven classification.

As part of the feasibility assessment, participants received weekly telephone check-ins conducted by trained members of the research team. These calls followed a semi-structured interview guide consisting of 12 standardized questions designed to capture participants’ experiences with the HELP platform. Questions assessed multiple domains, including overall satisfaction with the resources, perceived engagement and usability, emotional responses to the content, the clarity and appropriateness of module length, the use of platform features, perceived impacts on behavior change readiness, and the time required to engage with the resources. Feedback collected during these calls was intended to inform future refinements to the HELP platform and was not used to modify the intervention during the study period. Limited participation in weekly check-ins constrained the ability to quantitatively analyze standardized feedback measures, and qualitative findings should therefore be interpreted as descriptive rather than representative.

### 2.4. Statistical Analyses

Student *t*-tests evaluated within-individual (paired) and inter-group (unpaired) differences. Cohen’s d effect sizes were calculated (small ≤ 0.2, medium 0.2 > score < 0.8 or large ≥ 0.8; Cohen, 1994 [19]; Cohen, 2013 [20]) to describe the magnitude of differences in scores. Chi-square examined gender/diagnosis by engagement. Statistical significance was defined as *p* < 0.05, though analyses were exploratory and not intended to support inferential conclusions given the small sample size. All analyses were conducted using Microsoft^®^ Excel^®^ 2016 Version 16.0.5478.1002 (Microsoft Corporation, Redmond, WA, USA).

## 3. Results

### 3.1. Recruitment and Attrition

A total of 394 youth were screened for eligibility (Figure 1). Of these, 169 were ineligible, most commonly due to age (*n* = 62), eating disorder diagnosis (*n* = 42), or English-language limitations (*n* = 12.) Among the 163 youth determined to be eligible, 129 declined participation. The most cited reasons included being unresponsive (*n* = 70), a lack of interest (*n* = 25), time constraints (*n* = 10), skepticism about project utility (*n* = 10), and parental concerns of added stress (*n* = 4). In total, 34 youth consented and enrolled, representing 21% of those eligible. Of the 34 enrolled participants, 11 withdrew prior to completing baseline assessments. The reasons for pre-baseline withdrawal included loss to follow-up (*n* = 3), lack of interest (*n* = 2), no longer being eligible (*n* = 2), time constraints (*n* = 1), and complicated mental health issues (*n* = 1). Of the 23 youth who completed the baseline assessments, an additional 13 withdrew during the 3-month access period, most commonly due to loss to follow-up (*n* = 5), lack of interest/complicated mental health issues (*n* = 3), time constraints (*n* = 2), and no longer being eligible (*n* = 2). Participant recruitment and attrition are further detailed in Figure 1.

### 3.2. Engagement with HELP

Among the 10 participants who completed the baseline and 3-month follow-up, 7 (70%) accessed the HELP e-resources on more than 10 days during the 3-month access period. Five participants (50%) accessed each of the screen time, sleep, and physical activity modules on more than 10 days, although these were not necessarily the same individuals across domains. Three participants (30%) accessed all three behavior modules on more than 10 days, 2 (20%) accessed two domains, 2 (20%) accessed one domain, and 3 (30%) did not exceed 10 days of access for any single lifestyle behavior.

As not all participants completed all assessments at each time point, sample sizes vary across outcomes. Assessment completion by participant and time point is summarized in Supplementary Table S1.

### 3.3. Study Participants

Information regarding participant characteristics and diagnoses are shown in Table 1 for participants that completed the assessment and 3-month access periods. Age (*p* = 0.73), gender (*p* = 0.62), and diagnosis (*p* = 0.18) did not differ between participants who did or did not complete the study.

### 3.4. Participant Feedback

During the weekly check-ins conducted over the course of the intervention, participants provided both positive and constructive feedback regarding the program. Youth expressed appreciation for the motivational impact of goal setting and its positive effects on both them and family.

*“I have really enjoyed the resources I’ve used so far. They’ve been super detail and helpful in helping me find different ways to maintain a healthy lifestyle.”*.

(H17, Male, 18, Week 4 Check-In)

*“It makes me feel happy and my mom feel reassured that there is something good for me out there.”*.

(H11, Male, 14, Week 4 Check-In)

*“[I’m] definitely motivated, I feel like I have a way that can help me get towards doing better with this stuff.”*.

(H22, Female, 16, Week 4 Check-In)

*“I have liked them I really like being able to set goals and being given options of goals to choose from.”*.

(H19, Female, 15, Week 7 Check-In)

Even among those who did not complete the program, there were positive reflections. One participant initially shared that HELP

*“…made me feel discouraged at times”*.

(H25, Female, 13, Week 1 Check-In)

However, two weeks later, they also noted that

*“The goals, I like how you can set goals it gives you kind of step by step on how you achieve the goal”*.

(H25, Female, 13, Week 3 Check-In)

There was also constructive suggestions for improvement, with one noting that

*“I like the amount of resources that there are but sometimes I feel like they can be kind of long which can be overwhelming”*.

(H25, Female, 13, Week 3 Check-In)

While another suggested that

*“More things like that to make it personalized for the user would be great.”*.

(H17, Male, 18, Week 4 Check-In)

### 3.5. Baseline Differences by HELP Study Completion

**Figure 2:**
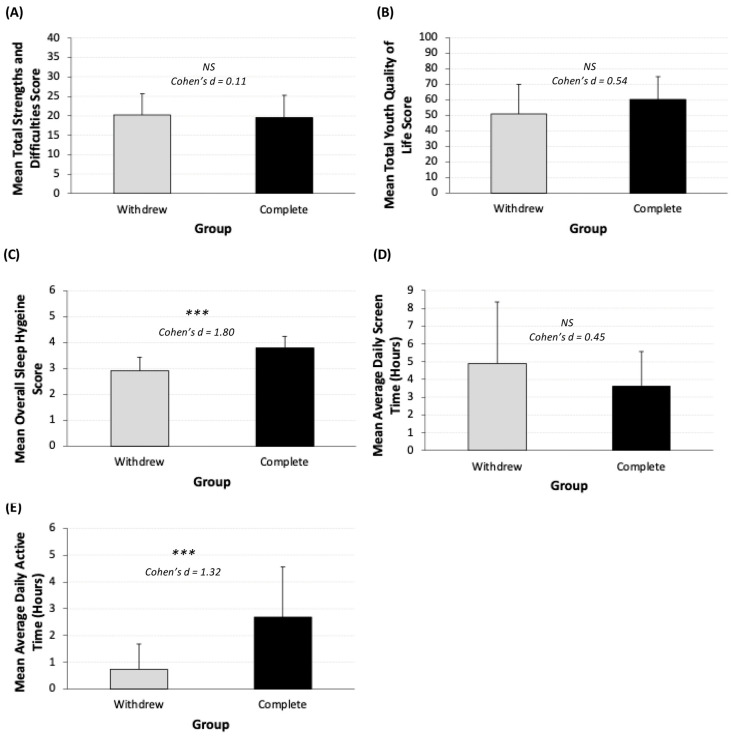
(**A**–**E**). Comparison of baseline scores by study completion/withdrawal. (**A**) Strengths and difficulties (total SDQ) (withdrew: *n* = 9; Completed: *n* = 10); (**B**) youth quality of life (withdrew: *n* = 9; complete: *n* = 10); (**C**) sleep hygiene score (withdrew: *n* = 13; completed: *n* = 10); (**D**) average daily screen time (withdrew: *n* = 10; completed: *n* = 10). (**E**) Average daily active hours (withdrew: *n* = 8; completed: *n* = 10); error bars represent standard deviation. Asterisks represent level of significance (*p* > 0.1 = NS, *p* < 0.05 = ***) . Sample sizes vary across analyses due to incomplete assessment completion. Participants who did not complete a given assessment were excluded from the analyses for that outcome.

Emotional and behavioral difficulties (SDQ total score) did not differ by adherence (completed: mean 19.6 ± 5.7 points; withdrew: mean 20.2 ± 5.5 points, *p* = 0.81; Cohen’s d = 0.11; Figure 2A). Quality of life (total YQoL score) was higher among adherent participants (completed: mean 60.1 ± 14.6 points; withdrew: mean 51.0 ± 18.9 points, *p* = 0.26; Cohen’s d = 0.54; Figure 2B). Adherent participants reported better sleep (total ASHS score; completed: mean 3.8 ± 0.4 points; withdrew: mean 2.9 ± 0.5 points, *p* < 0.001; Cohen’s d = 1.80; Figure 2C). Although not statistically significant, screen time was higher amongst withdrawn participants (completed: mean 3.6 ± 2.0 h/day,; withdrew: 4.9 ± 3.5 h/day, *p* = 0.33, Cohen’s d = 0.45; Figure 2D). Adherent participants also reported more active hours/day (completed: mean 2.7 ± 1.9; withdrew: mean 0.7 ± 1.0 h/day, *p* = 0.012, Cohens’ d = 1.32; Figure 2E).

### 3.6. Overall Impact of HELP Resource Access

**Figure 3:**
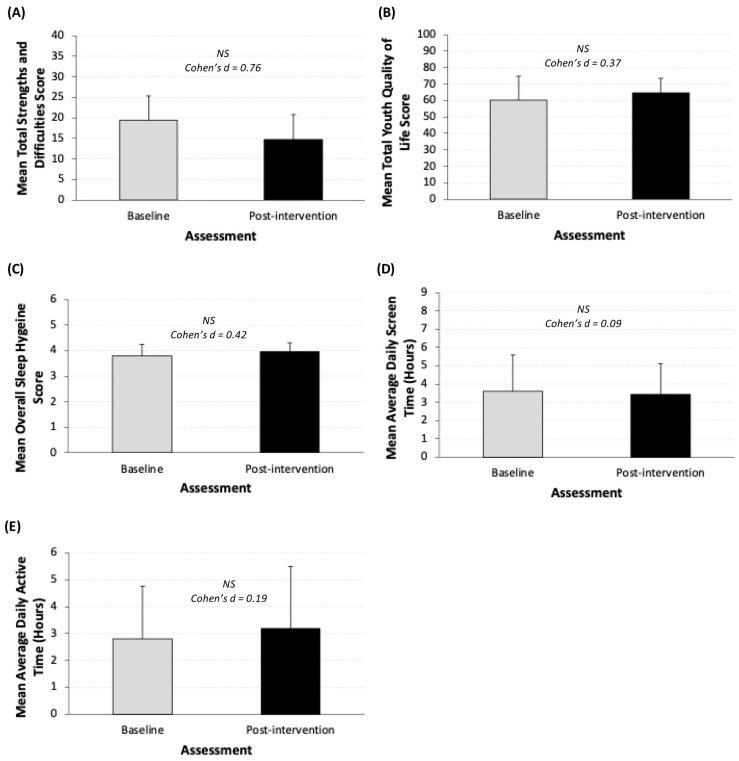
(**A**–**E**). Pre- and post-intervention comparison of outcome measures (*n* = 10). (**A**) Strengths and difficulties (total SDQ) ; (**B**) youth quality of life; (**C**) sleep hygiene; (**D**) average daily screen time; (**E**) average daily active hours; error bars represent standard deviation. Asterisks represent level of significance (*p* > 0.1 = NS). Sample sizes vary across analyses due to incomplete assessment completion. Participants who did not complete a given assessment were excluded from the analyses for that outcome. One participant did not complete the final Strengths and Difficulties and Habitual Activity Estimation Scale questionnaires. Baseline scores for this participant were also omitted. Findings should be interpreted with caution due to the very small sample size.

Study completion was associated with moderate effect size improvements in emotional and behavioral difficulties (pre-intervention: mean total SDQ score 19.3 ± 6.0 points; post-intervention: mean total SDQ score 14.8 ± 6.1 points, *p* = 0.13, Cohen’s d = 0.76; Figure 3A), quality of life (pre-intervention: mean total YQoL score 60.1 ± 14.6 points; post-intervention: mean total YQoL score 64.7 ± 8.9 points, *p* = 0.41, Cohen’s d = 0.37; Figure 3B), and sleep (pre-intervention: mean total ASHS score 3.8 ± 0.4 points; post-intervention: mean total ASHS score 4.0 ± 0.3 points, *p* = 0.36, Cohen’s d = 0.42; Figure 3C). Average daily screen time scores as measured by the ASAQ did not differ (pre-intervention: 3.6 ± 2.0 points; post-intervention: 3.4 ± 1.7 points, *p* = 0.84, Cohen’s d = 0.09; Figure 3D), and neither did average daily active hours as measured by the HAES (pre-intervention: 2.8 ± 2.0 h; post-intervention: 3.2 ± 2.3 h, *p* = 0.70, Cohen’s d = 0.19; Figure 3E).

### 3.7. Impact of HELP Resource Access by Engagement Level

**Figure 4:**
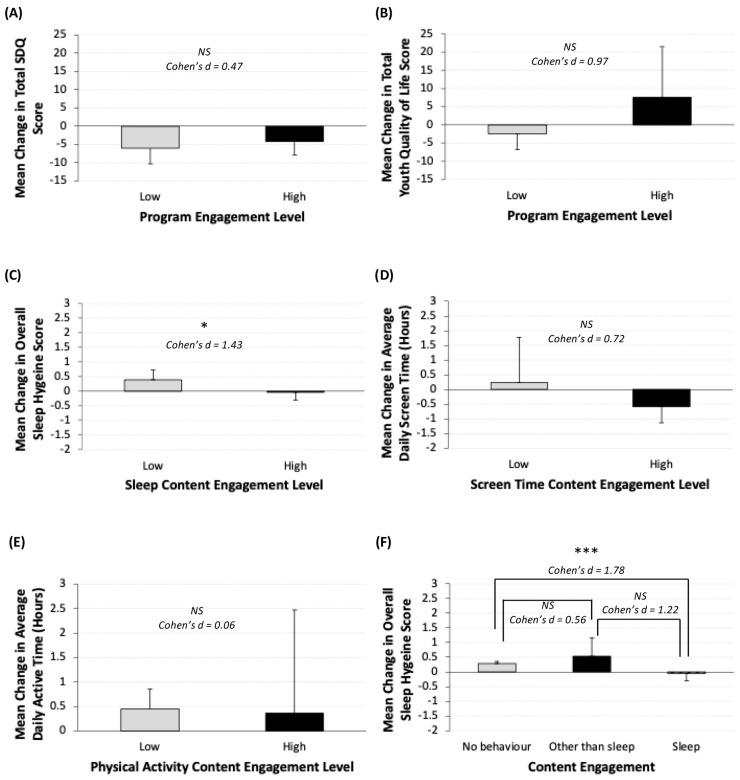
(**A**–**F**) Intervention impact by level of virtual resource engagement. (**A**) Strengths and difficulties (total SDQ) (low: *n* = 2; high: *n* = 7); (**B**) youth quality of life (low: *n* = 3; high: *n* = 7); (**C**) sleep hygiene (low: *n* = 5; high: *n* = 5); (**D**) average daily screen time (low: *n* = 5; high: *n* = 5); (**E**) average daily active hours (low: *n* = 4; high: *n* = 5); (**F**) sleep hygiene based on engagement with no behaviors (*n* = 3), engagement with other behaviors (*n* = 2) and engagement with both sleep and other behaviors (*n* = 5). Error bars represent standard deviation. Asterisks represent level of significance (*p* > 0.1 = NS, *p* < 0.1 = *, *p* < 0.05 = ***). Sample sizes vary across analyses due to incomplete assessment completion. Participants who did not complete a given assessment were excluded from the analyses for that outcome. Findings should be interpreted with caution due to the very small sample size.

Mean total SDQ score did not differ significantly by HELP website engagement (low: −6 ± 4.2 points; high: −4.1 ± 3.7 points, *p* = 0.67, Cohen’s d = 0.47; Figure 4A). However, participants with high levels of HELP website engagement tended to report greater improvements in quality of life as measured by the total YQoL score (low: −2.4 ± 4.3 points; high: 7.5 ± 13.9 points, *p* = 0.13, Cohen’s d = 0.97; Figure 4B), with a large effect size observed. Participants who were highly engaged with the sleep content experienced a stable total ASHS score (mean difference: −0.05 ± 0.3 points), while those with low engagement reported an improved score (mean difference: 0.4 ± 0.3 points). The difference in mean score change between these groups was not statistically significant, but a large effect size was observed (*p* = 0.06, Cohen’s d = 1.43, Figure 4C). Youth who engaged with screen time-specific content tended to report favorable changes in daily screen time as measured by the ASAQ, with a moderate effect size (low: screen time increased by 0.2 ± 1.5 h; high: screen time decreased by 0.6± 0.5 h, *p* = 0.31, Cohen’s d = 0.72; Figure 4D). Changes to daily active hours as measured by the HAES were unrelated to engagement (low: 0.45 ± 0.40 h; high: 0.36 ± 2.1 h, *p* = 0.93, Cohen’s d = 0.06; Figure 4E).

Among those with limited engagement with sleep content, participants who did not engage with any of the three lifestyle behaviors reported a small average increase in sleep hygiene (mean difference: 0.3 ± 0.07 points), and those who engaged with lifestyle behaviors other than sleep reported a slightly greater score improvement (mean difference: 0.5 ± 0.6 points). A moderate effect size was indicated (*p* = 0.67, Cohen’s d = 0.56, Figure 4F). Participants not engaged with any of the three lifestyle behaviors experienced a positive change in sleep hygiene as compared to those who engaged with sleep-related content (*p* = 0.04, Cohen’s d = 1.78). Similarly, those who engaged with lifestyle behaviors other than sleep experienced a greater improvement as compared to those who engaged with the sleep-related content (*p* = 0.42, Cohen’s d = 1.22)

## 4. Discussion

Promoting positive lifestyle changes has been shown to support psychological well-being, including decreased depression and anxiety [21], increased self-esteem [22], and improved emotional regulation [23]. Despite these benefits, youth experiencing mental distress often struggle to engage in healthy behaviors [24,25]. As a result, there is growing interest in scalable, community-based interventions that can support behavior change while youth await formal mental health care. This pilot study on HELP was designed as an early-phase, feasibility-focused assessment to explore whether youth experiencing mental distress could engage with a self-directed lifestyle intervention, and preliminary trends in mental health and lifestyle behaviors over the intervention period. Given the small effective sample size and reliance on self-reporting, all findings should be considered exploratory and observed change should be interpreted with caution.

### 4.1. Recruitment, Retention, and Feasibility

Within this study, recruitment and retention posed notable challenges, with only 21% of eligible youth agreeing to enroll and 29% of those completing the 3-month access period and follow-up assessments. These rates align with previous web-based intervention programs for adolescents with anxiety [26] and depression [27], which reported enrollment rates of approximately 16% and 21% of screened participants, respectively. In our study, ineligibility was the main barrier to enrollment, most commonly due to the young age of the patient or an eating disorder diagnosis. Limited staff availability also contributed to recruitment challenges, as a subset of eligible youth could not be contacted. Higher recruitment rates reported in other studies have occurred in school-based settings [28] or in interventions with fewer exclusion criteria [29]. To more rigorously evaluate HELP, future studies will need to improve recruitment numbers. This may be achieved through broader recruitment settings, as well as enhanced support and follow-up during onboarding.

Retention rates for self-directed youth mental health interventions vary, with some studies reporting higher retention [9] and others reporting lower rates [26]. Studies reporting higher retention rates involved shorter intervention periods than HELP. In our study, participants who withdrew tended to report poorer mental health at baseline. While this observation should be interpreted cautiously, it is consistent with findings reported by Manicavasgar et al., suggesting that youth with increased mental health challenges may be more likely to disengage from self-directed web interventions. This raises concerns of selective attrition, which may bias our results towards a healthier subgroup and limit the generalizability of the findings to youth with higher baseline levels of mental distress. In real-world settings, this may indicate that youth with milder mental health difficulties at baseline are more likely to engage with and derive benefits, while those with greater needs may require additional supports. Adherence and engagement rates have been shown to improve when participants receive professional treatment concurrent to web-based resource use [30]. HELP intentionally omitted professional support to assess how youth could benefit from the e-resources during the waiting period for specialized treatment. Interventions leveraging automated feedback to simulate human interaction may be helpful as they have demonstrated greater effectiveness as compared to those with no interaction [31,32,33]. Self-guided web-based interventions that included an element of supportive peer interactions amongst participants have also shown promise for enhancing adherence and outcomes [34]. Engagement strategies for youth with higher baselines distress, and alternative non-professional support mechanisms warrant further investigation to strengthen the feasibility and internal validity in future studies.

### 4.2. Exploratory Mental Health and Lifestyle Outcomes

Among the youth who completed the study, access to lifestyle behavior change e-resources showed early signs of potential benefit. SDQ scores improved from borderline (16–19 points) at baseline to normal (≤15 points) after the 3-month intervention period [35]. Participants who engaged with one or more lifestyle behaviors reported favorable trends in quality of life. Engagement with screen time content tended to be associated with reductions in self-reported daily screen time, and youth focusing on behaviors other than sleep reported suggestive improvements in sleep habits. Medium-to-large effect size estimates suggest that statistically significant improvements may be detectable in a larger, adequately powered study. No changes were observed in daily active time.

Lifestyle behaviors appeared to differ in their responsiveness to engagement with the HELP e-resources. While screen time habits appeared to be the most responsive to engagement, post-intervention screen time still exceeded the recommended limit of 2 h daily [36]. Nonetheless, prior research has shown that incremental reduction in screen time is associated with progressive improvement in depression and anxiety symptoms [37,38], supporting the continued inclusion of screen time reduction guidance within the HELP e-resources even when guideline targets are not fully achieved during the intervention timeframe.

Sleep behaviors appeared less directly responsive to engagement with HELP sleep-specific content. Previous studies have reported sleep duration as one of the strongest correlates of mental health indicators in adolescents [39] and young adults [40]. School-based sleep hygiene education programs have previously been successful in terms of improving sleep behaviors [41,42], yet participants who engaged with HELP sleep e-resources did not appear to report favorable changes in comparison to those focused on other lifestyle behaviors. Reduced screen time and physical activity have been shown to correlate with improved sleep quality in adolescents [43], raising the hypothesis that the observed trends in sleep hygiene may have occurred secondary to changes in other lifestyle behaviors rather than through direct engagement with sleep-specific content [44]. Mean sleep scores suggest that improvements in sleep hygiene were not limited by a ceiling effect. Future trials are needed to evaluate the specific impact of HELP sleep content and assess whether sleep outcomes are more effectively addressed through an integrated, multi-behavior approach.

In this pilot study, self-reported physical activity appeared least responsive to the HELP e-resources. It is unknown whether the limited observed change reflects a measurement limitation associated with reliance on self-report, or aspects of the e-resource content itself. While meeting the recommended 60 min of daily physical activity has been associated with progressively better mental health [37], in this study both pre- and post-intervention reports of average daily active time exceeded the recommended levels [36]. This suggests possible measurement limitations or a biased sample of highly active participants. Additionally, the existing literature has identified that youth engagement in physical activity is often greater in structured or group-based contexts [45]. This highlights the importance of considering activity types to maximize potential benefits. The HELP e-resource may benefit from exploring opportunities to encourage structured or group-based physical activities, including partnering with community or sport organizations.

In this study, participants were able to choose which behaviors to target. While adherence to multiple behaviors guidelines, including sleep, physical activity, and screen time, appears to yield greater mental health benefits [39], further research is required to determine whether encouraging participants to change across multiple lifestyle behaviors would be feasible and lead to more substantial improvements in mental health outcomes.

### 4.3. Strengths and Limitations

This preliminary study had several strengths and limitations. Approximately one fourth of enrolled youth completed the intervention, providing early insight into the feasibility of engaging youth with mental distress in a self-directed lifestyle intervention prior to receiving professional support. Emotional and behavioral difficulties scores and selected lifestyle behaviors appeared to be associated with patterns of improvement. While these observations are strictly preliminary, they suggest that HELP warrants further investigation as a potential support to bridge gaps in pediatric mental health care. Participant feedback indicated that HELP was positively regarded. One participant who initially expressed discouragement, later shared more positive reflections over time. It is important to note that participation in weekly check-ins was limited, precluding quantitative analysis. As such, the qualitative feedback should be interpreted as exploratory rather than representative of participant experience. The primary limitations of this study were the small sample size and substantial attrition. This limited statistical power and constrained the interpretation and generalizability of the observed trends. Differences were observed between participants who completed the study and those who withdrew, with non-completers demonstrating poorer baseline sleep hygiene and physical activity. This pattern raises concerns regarding selective attrition and potential bias towards youth with milder mental health difficulties. Given the absence of a control group, changes observed over the intervention period cannot be solely attributed to engagement with HELP e-resources. Behavioral outcomes were exclusively assessed using self-reported data, which may have been subject to bias. Together, these findings support the need for an appropriately powered study to further evaluate feasibility, engagement and effectiveness across diverse youth with experience of mental distress.

## 5. Conclusions

This pilot study revealed early insights into the implementation, engagement, and exploratory outcome patterns of the HELP virtual lifestyle behavior change e-resources among youth experiencing mental distress. The low recruitment and substantial attrition were important findings and highlight the challenges of delivering and evaluating a self-directed lifestyle intervention for youth with mental health challenges. Based on the suggestive trends of positive change among those who engaged, we hypothesize that the intervention may be most applicable to youth who report fewer emotional and behavioral difficulties and mild deficits in quality of life. However, given the limitations of the study, neither feasibility nor intervention effects could be confirmed, and all outcomes patterns must be considered exploratory. Nonetheless, these observations will inform the design of a larger- controlled trial warranted to more thoroughly assess feasibility, and the potential effects of the HELP e-resources on the emotional and behavioral difficulties, quality of life, and lifestyle behaviors of youth.

## Figures and Tables

**Table 1:** Participants baseline age, gender, and diagnosis: by total sample and by HELP program adherence.

	Total (*n* = 23)		Complete Data (*n* = 10)		Withdrawn (*n* = 13)	
	*M*	*SD*	*M*	*SD*	*M*	*SD*
Age (years)	15.7	1.7	15.8	1.2	15.5	2.1
	*n*	%	*n*	%	*n*	%
Gender						
Female	14	61%	7	70%	7	54%
Male	7	30%	3	30%	4	31%
Non Binary	1	4%	0	0%	1	8%
Unreported	1	4%	0	0%	1	8%
	*n*	%	*n*	%	*n*	%
Patient Diagnoses †						
Anxiety	12	52%	6	60%	6	46%
Neurodevelopmental Disorder	9	39%	5	50%	3	23%
Depression	8	35%	5	50%	4	31%
Diagnosis Unavailable	7	30%	2	20%	5	38%

Note: No inter group differences (*p* > 0.17). Abbreviations: *M* mean, *SD* standard deviation. † Diagnoses were those specified in the medical chart. Many participants had comorbid diagnoses; therefore, the total exceeds the number of participants.

## Data Availability

The raw data supporting the conclusions of this article will be made available by the authors on request. The data are not publicly available due to privacy restrictions for children receiving health services.

## Supplementary Materials

The following supporting information can be downloaded at: https://www.mdpi.com/article/10.3390/children13020163/s1, Table S1: Questionnaire completion by participant and time point.
