# Supplementary material for: Enhancing Youth Mental Health Through Virtual Lifestyle Behavior Change Support: A Pilot Feasibility Trial
Source: Children (Basel). 2026 Jan 23;13(2):163. doi: 10.3390/children13020163 (PMC12940004; doi:10.3390/children13020163)
Supplement: Supplementary file 1 [file children-13-00163-s001.zip › children-4008840-supplementary.pdf]

SUPPLEMENTARY

Supplementary Table S1. Questionnaire completion by participant and time point.

	BASELINE					3-MONTH				
Record ID	*ASHS*	*ASAQ*	*YQoL*	*SDQ*	*HAES*	*ASHS*	*ASAQ*	*YQoL*	*SDQ*	*HAES*
H01	I	I	I	I	I	I	I	I	I	I
H02	C	C	C	C	C	C	C	C	I	I
H03	I	I	I	I	I	I	I	I	I	I
H04	C	P	I	I	I	I	I	I	I	I
H05	C	C	C	C	C	C	P	I	I	I
H06	I	I	I	I	I	I	I	I	I	I
H07	C	C	C	C	C	C	C	C	C	C
H08	I	I	I	I	I	I	I	I	I	I
H09	C	C	C	C	C	I	I	I	I	I
H10	I	I	I	I	I	I	I	I	I	I
H11	C	C	C	C	C	C	C	C	C	C
H12	I	I	I	I	I	I	I	I	I	I
H13	C	I	I	I	I	I	I	I	I	I
H14	C	C	C	C	C	I	I	I	I	I
H15	I	I	I	I	I	I	I	I	I	I
H16	I	I	I	I	I	I	I	I	I	I
H17	C	C	C	C	C	C	C	C	C	C
H18	C	C	C	C	C	C	C	C	C	C
H19	C	C	C	C	C	C	C	C	C	C
H20	C	P	I	I	I	I	I	I	I	I
H21	C	C	C	C	C	I	I	I	I	I
H22	C	C	C	C	C	C	C	C	C	C
H23	C	C	C	C	C	C	C	C	C	C
H24	C	C	C	C	C	C	C	C	C	C
H25	C	C	C	C	C	I	I	I	I	I
H26	I	I	I	I	I	I	I	I	I	I
H27	C	C	C	C	C	C	C	C	C	C
H28	I	I	I	I	I	I	I	I	I	I
H29	I	I	I	I	I	I	I	I	I	I
H31	I	I	I	I	I	I	I	I	I	I
H32	C	C	C	C	C	I	I	I	I	I
H33	C	C	C	C	C	I	I	I	I	I
H34	C	C	C	C	C	I	I	I	I	I
H35	C	C	C	C	C	I	I	I	I	I
H36	I	I	I	I	I	I	I	I	I	I
H37	C	I	I	I	I	I	I	I	I	I
H38	C	C	C	C	C	C	C	C	C	C
H39	C	C	C	C	I	I	I	I	I	I

C = complete; P = partially complete; I = incomplete. Cells shaded in grey indicate questionnaires that were not included in analyses (P or I). Only fully completed questionnaires were included in analyses. Participants H05 and H18 were excluded from all analyses as they did not meet study eligibility criteria.
